# Instruction in the True Principles of Homœopathy

**Published:** 1891-02

**Authors:** 


### INSTRUCTION IN THE TRUE PRINCIPLES OF HOMŒOPATHY.

In no other branch of human endeavor is the saying truer than in Homœopathy, that one must start right in order to end right; the same thought is often expressed in the maxim, "Well-begun is half done." In the practice of Homœopathy one must know its philosophy, as taught by Hahnemann and his true disciples, if he would be successful in curing the sick. This knowledge is to be acquired before one can properly use the materia medica, or perform any clinical duty; the theory must be studied before practice can be undertaken. A correct understanding of the true principles which are to govern the physician in his clinical work is vastly more important than the memorizing of the materia medica. A physician who understands thoroughly the true principles of Homœopathy, and has at the same time a scant knowledge of the materia medica, will be more apt to prescribe correctly than one who knows all the symptoms of all the remedies, from Aconite to Zinc, but is ignorant of the true principles which are to guide him in administering those remedies.

A mere knowledge of the many indications for the use of our remedies does not teach one how to use them. Putting a box of tools into a man's hands does not make him a carpenter, nor does the putting of our materia medica into a student's head make him a physician; in both cases the tyro must be taught how to use the tools. In the education of our students too much attention has been given to a description of our tools and too little time given to training the student in the use of them. Suppose a student is perfectly trained as to when to give a remedy, Aconite for example; is he a well-trained physician unless he also knows how to give it, when to stop giving it, when to repeat the dose, or when to change the remedy?

As little attention is paid to this branch of homœopathic teaching in our colleges, the student must in general gain this knowledge for himself, and fortunate is he if he knows how and where to obtain it. Our literature upon this subject is confined to the writings of Hahnemann and to many miscellaneous essays by his disciples. The writings of the late Carroll Dunham are, or should be, familiar to all students of Homœopathy. Hering, Lippe, and others have written many invaluable essays at different times and published in different places; but no follower of Hahnemann has written more wisely and in such detail as our venerable friend, Dr. P. P. Wells. It is the purpose of this article to give a brief list of some of his essays which treat directly of the many points involved in a true knowledge of homœopathic medicine.

As these essays have been published in THE HOMŒOPATHIC PHYSICIAN during the past ten years, they are all, doubtless, familiar to the readers of this journal. But the following list is arranged in such order as to treat of the prominent principles of Homœopathy in a sequence of subjects; each essay, partially at least, continuing and completing the topic considered in the previous one. In this order, these essays will doubtless appear in a new light to those well acquainted with them in their previous scattered state. It will seem obvious to any one that these essays, to a great extent, fill out a complete text-book upon the principles of true homœopathic practice; treating of its philosophy, of its materia medica, of how to choose and how to give the true remedy.

1. The Philosophy of Homœopathy.
2. What is Homœopathic Prescribing?
3. What Shall we Treat?
4. What is the Best Method of Selecting the Remedy?
5. Homœopathic Therapeutics and Pathological Anatomy.
6. Specific Prescribing as against Pathological Prescribing.
7. The Philosophy of the Materia Medica, its Study and its Uses.
8. The Materia Medica—A Science, its Nature, Uses, and How to Use it.
9. The Materia Medica and its Practical Uses.
10. Differentiation of Remedies; using Aconite, Belladonna, and Bryonia as Examples.
11. Errors in Drug Proving.
12. The Management of the Specific Remedy.
13. The Single Remedy.
14. Repetition of the Dose.
15. Alternation of Remedies.
16. High Potencies, Have they Efficient Action on the Organism?
17. Practical Surgery.
18. Hahnemann's Chronic Miasms.
19. Cholera, Snake Bite and their Lessons.
20. Hydrophobia, Prevention and Cure.

As stated before, these essays almost make in themselves a complete text-book upon the philosophy and practice of Homœopathy. Would it not, therefore, be beneficial to the profession to have them republished in a convenient book form? Would they not serve alike to freshen up the old practitioner and to teach the young physician? Would not this little volume serve as an excellent introduction to the study of Homœopathy? These essays could, doubtless, be printed in good style for, say, a dollar a volume. Will the readers of this journal each take a copy and so help to issue a good missionary volume?

There is no reader of this journal, however experienced and learned he may be, who would not be a more thorough Hahnemannian and a more accurate prescriber were he to carefully study over these essays. If these essays can be republished in book form, our school will have better *instruction in the true principles of Homœopathy.* Will you help?

